# Proteotyping of *Clostridioides difficile* as Alternate Typing Method to Ribotyping Is Able to Distinguish the Ribotypes RT027 and RT176 From Other Ribotypes

**DOI:** 10.3389/fmicb.2019.02087

**Published:** 2019-09-10

**Authors:** Matthias F. Emele, Felix M. Joppe, Thomas Riedel, Jörg Overmann, Maja Rupnik, Paul Cooper, R. Lia Kusumawati, Fabian K. Berger, Friederike Laukien, Ortrud Zimmermann, Wolfgang Bohne, Uwe Groß, Oliver Bader, Andreas E. Zautner

**Affiliations:** ^1^Institut für Medizinische Mikrobiologie, Universitätsmedizin Göttingen, Göttingen, Germany; ^2^Leibniz-Institut DSMZ-Deutsche Sammlung von Mikroorganismen und Zellkulturen GmbH, Braunschweig, Germany; ^3^Deutsches Zentrum für Infektionsforschung (DZIF), Standort Hannover-Braunschweig, Braunschweig, Germany; ^4^National Laboratory for Health, Environment and Food (NLZOH), Maribor, Slovenia; ^5^Faculty of Medicine, University of Maribor, Maribor, Slovenia; ^6^St. Martin de Porres Hospital, Eikwe, Ghana; ^7^Department of Microbiology, Faculty of Medicine, Universitas Sumatera Utara, Medan, Indonesia; ^8^National Reference Center for Clostridioides (Clostridium) difficile, Institute of Medical Microbiology and Hygiene, Saarland University, Homburg, Germany

**Keywords:** MALDI-TOF MS, *Clostridioides difficile*, *Clostridium difficile*, below species differentiation, proteotyping

## Abstract

*Clostridioides difficile*, a Gram-positive spore-forming bacterium, is the leading cause of nosocomial diarrhea worldwide and therefore a substantial burden to the healthcare system. During the past decade, hypervirulent PCR-ribotypes (RT) e.g., RT027 or RT176 emerged rapidly all over the world, associated with both, increased severity and mortality rates. It is thus of great importance to identify epidemic strains such as RT027 and RT176 as fast as possible. While commonly used diagnostic methods, e.g., multilocus sequence typing (MLST) or PCR-ribotyping, are time-consuming, proteotyping offers a fast, inexpensive, and reliable alternative solution. In this study, we established a MALDI-TOF-based typing scheme for *C. difficile.* A total of 109 ribotyped strains representative for five MLST clades were analyzed by MALDI-TOF. MLST, based on whole genome sequences, and PCR-ribotyping were used as reference methods. Isoforms of MS-detectable biomarkers, typically ribosomal proteins, were related with the deduced amino acid sequences and added to the *C. difficile* proteotyping scheme. In total, we were able to associate nine biomarkers with their encoding genes and include them in our proteotyping scheme. The discriminatory capacity of the *C. difficile* proteotyping scheme was mainly based on isoforms of L28-M (2 main isoforms), L35-M (4 main isoforms), and S20-M (2 main isoforms) giving rise to at least 16 proteotyping-derived types. In our test population, five of these 16 proteotyping-derived types were detected. These five proteotyping-derived types did not correspond exactly to the included five MLST-based *C. difficile* clades, nevertheless the subtyping depth of both methods was equivalent. Most importantly, proteotyping-derived clade B contained only isolates of the hypervirulent RT027 and RT176. Proteotyping is a stable and easy-to-perform intraspecies typing method and a promising alternative to currently used molecular techniques. It is possible to distinguish the group of RT027 and RT176 isolates from non-RT027/non-RT176 isolates using proteotyping, providing a valuable diagnostic tool.

## Introduction

*Clostridioides difficile* (Lawson et al., 2016) is a Gram-positive anaerobic spore former and the most frequent cause of antibiotic-associated diarrhea (Lo Vecchio and Zacur, 2012; Leffler and Lamont, 2015; Martin et al., 2016; Smits et al., 2016). Current research revealed that this pathogen is responsible for more than 152,000 reported healthcare-associated *C. difficile* infections and more than 8,300 associated deaths every year in the European Union and European Economic Area (EU/EEA) (Cassini et al., 2016). The incidence rate observed in the United States was consistent with the European one (Martin et al., 2016). The symptoms of a *C. difficile* infection (CDI) appear in various manifestations: The spectrum comprises rather weak symptoms like mild diarrhea but also serious forms like toxic megacolon, pseudomembranous colitis (PMC) or perforation of the colon (Nanwa et al., 2015). Although the potential for severe disease is high, most of the colonized individuals do not show any symptoms (Donskey et al., 2015; Elliott et al., 2017). Despite the fact that the involvement of the small intestine has been observed, characteristic PMC lesions are usually limited to the colon (Jacobs et al., 2001; Keel and Songer, 2006). Infections outside of the intestine only occur very rarely (Byl et al., 1996).

Over the last decade, different “hypervirulent” *C. difficile* strains emerged. The most prominent of these “hypervirulent” strains has been categorized as PCR-ribotype 027 (RT027), which has emerged especially in Canada, North America, and various European countries (Pépin et al., 2004; Loo et al., 2005; McDonald et al., 2005; Brazier et al., 2008; Indra et al., 2008; Valiente et al., 2014). Outbreak studies from these and other countries all over the world revealed that RT027 is associated with an intensification of the worldwide epidemic of nosocomial *C. difficile* infections, resulting in recurrent infections and high mortality rates (Loo et al., 2005; Hubert et al., 2007; Mooney, 2007; Redelings et al., 2007). Furthermore, recent studies point out that RT027 strains continue to contribute significantly to CDI incidence (He et al., 2013; Arvand et al., 2014; Arvand and Bettge-Weller, 2016).

Besides adherence and motility factors, virulence of *C. difficile* mainly depends on toxins encoded by the pathogenicity locus (PaLoc) (Carter et al., 2015).

Another toxin expressed by some *C. difficile* strains like the “hypervirulent” RT027 which is not encoded by the PaLoc is the binary toxin or *C. difficile transferase* (CDT) (Stubbs et al., 2000; Sundriyal et al., 2010). Although this toxin is supposed to enhance virulence and some studies show a correlation between its presence and an increased mortality rate, its exact role is unknown so far (Barbut et al., 2005; McEllistrem et al., 2005; Gerding et al., 2014). For a current overview of the *C. difficile* toxins we refer to the article of Klaus Aktories, Carsten Schwan, and Thomas Jank (Aktories et al., 2017).

There are several possible reasons for the spreading of RT027 strains: One reason is, that these strains show a higher resistance to fluoroquinolones compared to other strains (Sebaihia et al., 2006; Drudy et al., 2007; Dannheim et al., 2017) besides resistance to tetracycline, aminoglycosides, and erythromycin (Knetsch et al., 2018).

Another factor that possibly contributed to the spreading is the implementation of trehalose as a food additive, which came into the market shortly before the rise of virulent strains like RT078 and RT027. RT027 strains exhibit a single point mutation in the trehalose repressor what leads to a more than 500-fold increase of sensitivity to trehalose. Trehalose also increased the virulence of RT027 strains in mouse models of CDI (Robinson et al., 2014; Collins et al., 2018). However, recent studies could not find any association between trehalose metabolism variants and severe disease outcomes (Eyre et al., 2019).

Moreover, it was proposed that after antibiotic treatment, the re-colonization of the gut by commensals is inhibited by a phenol derivate, *p*-cresol, produced by *C. difficile* (Dawson et al., 2008). Also the ability to form spores has been proposed to contribute to the difference in virulence between RT027 and other *C. difficile* strains (Burns et al., 2010; Lanis et al., 2010).

There is a wide range of diagnostic methods available to investigate on the phylogeny of *C. difficile*, including PCR-ribotyping and multi locus sequence typing (MLST) (Griffiths et al., 2010; Knetsch et al., 2013). The most common method in Europe is PCR-ribotyping that now also applies to the United States (Janezic and Rupnik, 2010; Waslawski et al., 2013; Fawley et al., 2015). This approach was first described by Gürtler (1993) and makes use of length differences (200–600 bp) of the intergenic spacer region (ISR) between 16S and 23S rRNA genes. Furthermore, different *C. difficile* strains also exhibit different numbers of alleles in the ribosomal operon. By combining ISR length- and allele number variation, a specific banding pattern can be obtained for the respective ribotype by PCR amplification with a single primer pair (Janezic, 2016).

In contrast, MLST discriminates isolates using nucleotide sequences of housekeeping gene fragments (Maiden et al., 1998), where a sequence type (ST) number is assigned to each unique combination of alleles. The MLST technique is also scalable to high-throughput robotic systems (Pavón and Maiden, 2009).

To respond immediately in case of a disease outbreak, fast, accurate and inexpensive diagnostic methods are indispensable. Since PCR-ribotyping and MLST are relatively expensive and time-consuming, matrix-assisted laser desorption/ionization mass spectrometry (MALDI-TOF MS) represents a promising alternative (Lavigne et al., 2013; Patel, 2015). This technique has become the current standard for species identification in many clinical microbiological laboratories in many countries (Seng et al., 2010; Bader, 2013). Beside species identification, MALDI-TOF MS allows distinction of subspecies by accurate discrimination of strain-specific biomarkers (Lartigue, 2013; Suarez et al., 2013; Durighello et al., 2014). Previous studies have shown the possibility to differentiate *Salmonella enterica* ssp. *Enterica serovar Typhi* from *Salmonella enterica* ssp. *enterica* serotypes, which are of minor clinical relevance (Kuhns et al., 2012). Moreover, it was shown that it is even possible to discriminate different MLST sequence types (STs) of *Campylobacter jejuni* ssp. *jejuni* using a single biomarker ion (Zautner et al., 2013). Cheng et al. (2018) recently discovered that it is possible to differentiate Clade 4 strains of *C. difficile* from other *C. difficile* strains by MALDI-TOF MS on the basis of 5 markers. In another recent study, Corver and coworkers identified two peptide markers (*m/z* = 4927.81 and *m/z* = 5001.84) that enable the identification of *C. difficile* MLST types 1 and 11 by MALDI-MS (Corver et al., 2018). Another MALDI-TOF MS-based subtyping approach was published by Ortega and coworkers: They used a technique called high molecular weight (HMW) typing where a protein profile within the mass range of 30 to 50 kDa was analyzed (Rizzardi and Åkerlund, 2015; Ortega et al., 2018). More precisely this method groups *C. difficile* strains according to proteins of their surface layers. Within the study, they identified different HMW profiles. One of those profiles only harbors RT027 strains, what makes it an interesting tool for rapid subtyping (Ortega et al., 2018).

The main problem of clustering-based MALDI-TOF MS-typing methods is the lack of knowledge about the proteins that correspond to the respective peaks in the mass spectrum. This problem can be solved to a certain degree using proteotyping. This microbial typing method that we initially named Mass Spectrometry-based PhyloProteomics (MSPP), but which we will refer to as proteotyping, in accordance with the terminology now used in the scientific community (Karlsson et al., 2015), was previously successfully used for subtyping of *C. jejuni* ssp. *jejuni*, *C. jejuni* ssp. *doylei* and *Campylobacter coli* isolates (Zautner et al., 2015, 2016; Emele et al., 2019). The essential characteristic of our proteotyping method is an amino acid sequence catalog of isoforms of alleles. These isoforms are the result of non-synonymous mutations in genes coding for ribosomal proteins (biomarker genes). These mutations can be detected in the form of mass shifts within MALDI-TOF spectra. It is then possible to assign an isolate to a specific proteotyping-derived type by analyzing the scheme of recorded biomarker masses and deducing the respective amino acid sequence. The key advantage of proteotyping in comparison to whole mass spectrum clustering approaches is that only mass changes assigned to a specific set of allelic isoforms of the same protein are considered for deduction of phylogeny. Alternative methods that focus on presence or absence of single masses as well as peak intensity are leading to imprecise results (Suarez et al., 2013; Zautner et al., 2013; Matsumura et al., 2014; Novais et al., 2014).

For this study, we compiled a collection of 109 *C. difficile* strains to develop and test a *C. difficile-*specific proteotyping scheme.

## Materials and Methods

### *Clostridioides difficile* Isolates

In total, 109 *C. difficile* isolates were chosen in a way, that the test collection represented a high genetic diversity and the currently clinical relevant and most prevalent five out of eight established clades of this species (Stabler et al., 2009; Knetsch et al., 2012; Dingle et al., 2014; Knight et al., 2015; Riedel et al., 2017). For the MALDI-TOF analyses, 77 isolates were selected for which a complete genome was already sequenced (data not shown). More precisely, 46 clade 1 strains, 24 clade 2 strains, 2 clade 3 strains, 17 clade 4 strains and 5 clade 5 strains were selected for the experiments (Supplementary Table 1). To broaden the basis for the differentiation of RT027 isolates, additionally, 17 RT027, 10 RT176, 3 RT153, and 2 RT016 isolates were included in the study for which no genomic data was available. Isolates of clade C-I – III were not available and were not included in the study. The entire collection consisted of clinical isolates from four different countries: Germany, Great Britain, Ghana, and Indonesia (Seugendo et al., 2018).

### Bacterial Culture Conditions

*Clostridioides difficile* isolates were kept in store as cryobank stocks (Mast Diagnostica, Reinfeld, Germany) maintained at −80°C. Isolates were incubated for 48 h at 37°C on Columbia agar (Merck, Darmstadt, Germany) supplemented with 5% sheep blood (Oxoid, Wesel, Germany) under anaerobic condition using a COY anaerobic gas chamber (COY Laboratory Products, United States). The atmosphere used consisted of 85% N_2_, 10% H_2_, 5% CO_2_. All experiments were carried out under biosafety level 2 conditions.

### Preparation of Matrix Solution

To prepare the matrix solution used for the experiments α-cyano-4-hydroxy-cinnamic acid (HCCA) purified matrix substance (Bruker Daltonics, Bremen, Germany) was dissolved in standard solvent consisting of 47.5% MALDI-grade water, 50% acetonitrile, and 2.5% trifluoroacetic acid (all Sigma-Aldrich, Taufkirchen, Germany) by what the solution had a final concentration of 10 mg HCCA/mL. In order to have an internal calibrant for the measurements purified recombinant human insulin (Sigma-Aldrich, Taufkirchen, Germany) was added to HCCA. Human insulin was dissolved in 50% aqueous acetonitrile to attain a final concentration of 10 pg/μL. The precise determination of the insulin peak mass was done experimentally by mixing with Biotyper Test Standard (BTS, Bruker Daltonics) and yielded an *m/z* of 5,806.1. The insulin peak was chosen as internal calibrant for all *C. difficile* mass spectra because it did not cover any biomarker masses of interest. An internal calibrant has a crucial effect on precision during determination of biomarker mass variations. This approach enabled us to detect mass difference with an accuracy of up to 1 Da.

### MALDI-TOF Mass Spectrometry

Sample preparation for MALDI-TOF MS measurements was done using two different procedures following the manufacturer’s instructions: (i) smear preparation, which, from experience, allows a better detection of peaks in the *m/z* range >10,000 kDa and (ii) formic acid/acetonitrile extraction, facilitating more precise analysis in the *m/z* range <10,000 kDa.

Briefly, to prepare extract samples, five colonies that were plated for 48 h on agar were thoroughly resuspended in 300 μL ddH_2_O followed by the addition of 900 μL of absolute ethanol. The suspension was then mixed by pipetting up and down repeatedly. After complete suspension of the bacterial colonies the suspensions were centrifuged for 1 min (13,000 × *g*). The supernatant was discarded followed by drying of the pellets for approx. 10 min at room temperature. To resuspend the pellet in 50 μL of 70% formic acid it was vortexed thoroughly. 50 μL of acetonitrile were then added to each sample and again mixed by pipetting as previously described (Zautner et al., 2015), followed by centrifugation of the mixture for 2 min (13,000 × *g*) removing cellular debris. Subsequently, 1 μL of the supernatant was transferred into the designated field on a MALDI target plate, consisting of polished steel. It was left to dry at room temperature for approx. 5 min and subsequently overlaid with 1 μL of HCCA matrix containing the human insulin. After another drying step at room temperature samples were ready for MS-analysis.

MALDI-TOF MS measurements were performed according to the MALDI Biotyper standard procedures (Bruker Daltonics, Bremen, Germany). During analysis, 600 spectra in a mass range between 2 and 20 kDa were collected in 100-shots steps on an Autoflex III system and summed up. Results obtained with MALDI Biotyper (database release 2016) identification score values ≥2.000 were considered correct.

### Identification of Biomarkers in Mass Spectra

To analyze the received mass spectra, the software FlexAnalysis (Bruker Daltonics, Bremen, Germany) and its embedded standard algorithms were used. First, spectra were internally calibrated according to the known insulin peak (*m/z* = 5,806.1), followed by baseline subtraction (TopHat) and smoothing as implemented in the standard MBT method.

To determine the theoretical average molecular weight of the ribosomal proteins corresponding to the respective open reading frame of the different genomes (data not shown), the deduced amino acid sequences were uploaded separately to the molecular weight calculator tool at the ExPASy Bioinformatics Resource Portal^1^. Eukaryotic as well as ribosomal proteins of Enterobacteriaceae frequently undergo post-translational modifications (Gonzales and Robert-Baudouy, 1996; Varland et al., 2015). Consequently, further potential molecular weights needed to be calculated for each biomarker. In our context, the most relevant post-translational modification was the proteolytic removal of *N*-terminal methionine, which was considered with a mass difference of −131.04 Da. In addition to the cleavage of the *N*-terminal methionine, further post-translational modifications may occur, e.g., acetylation, phosphorylation, formylation, and methylation (Ouidir et al., 2015; Kentache et al., 2016).

In order to identify biomarker masses, more precisely to assign a calculated biomarker mass to a certain allelic isoform, measured masses were checked against the calculated masses of the *C. difficile* 630 (= DSM 27543) reference genome (Dannheim et al., 2017). If there was no clear correspondence between biomarker mass in the spectrum of a particular clinical isolate and the masses calculated from the *C. difficile* 630 (= DSM 27543) reference genome (GenBank Acc. No. CP010905.2), the spectrum was examined regarding peaks with a different molecular weight or more specifically amino acid substitutions that could be causal for the mass shift. Allelic isoforms in the test cohort were reconfirmed by *in silico* translation of the gene sequences taken from the complete bacterial genome and subsequent alignment of the resulting amino acid sequences. For each of the cases the predicted amino acid exchanges could be confirmed, which also served as additional argument in favor of the identity of the peak. Due to a lack of instrumentation the identity of the peaks was not re-confirmed by tandem mass spectrometry (MS/MS), which is only a minor uncertainty as the detection of ribosomal proteins in the corresponding mass range using MALDI-TOF MS with a Bruker biotype has been sufficiently demonstrated (Ryzhov and Fenselau, 2001; Dieckmann et al., 2008; Seng et al., 2010; Sandrin et al., 2013).

### Phylogenetic Analysis and Proteotyping

For handling of trace data, nucleotide sequences, and subsequent alignment of the deduced protein sequences, Geneious V 11.1.2, the Molecular Biology and NGS Analysis Tool was used (Biomatters Ltd., Auckland, New Zealand). For each biomarker (ribosomal protein encoding gene) identified in strain *C. difficile* 630 (= DSM 27543), the sequences were screened against the respective genome sequence of the 77 isolates for which genome sequence data was available. Subsequently, an amino acid sequence list containing all allelic isoforms of the 9 biomarkers included in the proteotyping scheme was assembled. To construct the unweighted pair group method using average linkages (UPGMA)-tree, Molecular Evolutionary Genetics Analysis X (MEGA X) software was used (Kumar et al., 2018).

The respective PCR-ribotypes of the isolates were determined by agarose (isolates from Indonesia and Ghana) or capillary gel electrophoresis (isolates from Germany) following consensus protocols (ECDIS-Net, CDRN) described in previous publications (Janezic and Rupnik, 2010; Fawley et al., 2016; van Dorp et al., 2016; Berger et al., 2018).

For the 17 isolates without genome sequence data, the gene loci for the ribosomal proteins L28 and L35 were sequenced using the following primers: CdiffL28-F01: 5′-GTT-ATC-ATT-TTA-AGG-AGG-TGT-GCG-3′ and CdiffL28-R01: 5′-TGG-C TG-GAT-TTG-GTC-AGC-AC-3′; CdiffL35-F01: 5′-ACC-AAC-AAA-AGC-CCC-TGC-AT-3′ and CdiffL35-R01: 5′-TCT-TGC-CAT-CGT-TAT-GAC-CTC-C-3′. PCR-reactions were conducted with the following parameters: two denaturation steps at 95°C for 30 s; annealing at 60°C for 1 min; two elongation steps at 68°C for 1 and 5 min. Sanger sequencing of the amplificates was performed by SeqLab-Microsynth (Göttingen, Germany).

## Results

**FIGURE 1:**
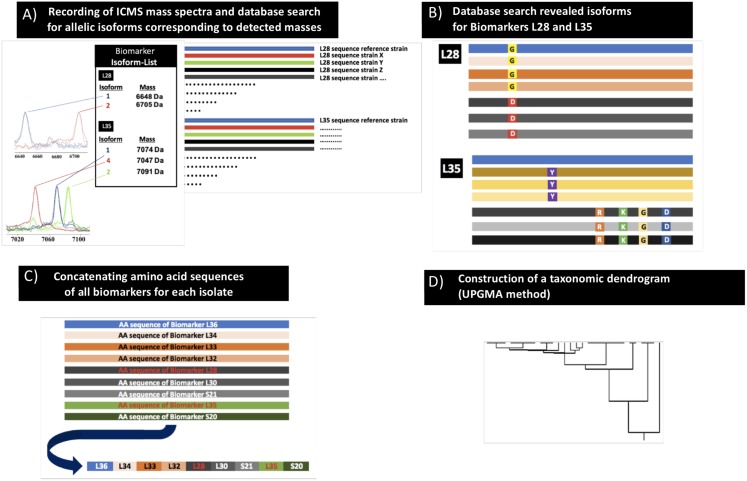
Scheme of the proteotyping workflow. **(A)** Recording of MALDI-TOF mass spectra of *C. difficile* isolates (extracts as well as smear preparation). **(B)** Identification of allelic isoforms by comparison with the allelic isoform database that contains the sequence data of the *C. difficile* genomes deposited in public databases. **(C)** Assembly of the concatenated amino acid sequences of the respective isoforms to one continuous sequence. **(D)** Calculation of a taxonomic proteotyping-derived UPGMA dendrogram.

The previously established proteotyping (MSPP) workflow (Zautner et al., 2015) was used to develop a *C. difficile*-specific proteotyping scheme as outlined in detail below (Figure 1). In summary, the mass spectrum of the genome sequenced *C. difficile* reference strain 630 (= DSM 27543) was recorded followed by the assignment of spectrum masses to protein-coding genes. Analysis of genome sequences received from the NCBI database enabled the establishment of an allelic isoforms list of the assignable spectrum masses. For all isolates included in the study, observed mass shifts in comparison to the spectrum of the *C. difficile* reference strain 630 (= DSM 27543) were noted and the allelic isoforms assigned by comparing observed mass shifts with the established isoform list. A proteotyping-based phyloproteomic tree was calculated from concatenated biomarker amino acid sequences (as required by the MEGA X software) and compared to the respective MLST data constructed in an analogous fashion.

### Identification of Reference Biomarker Ions

The initial step of the proteotyping workflow was the measurement of *C. difficile* reference strain 630 (= DSM 27543). The reproducibility of the MALDI-TOF mass spectra was sufficiently high. The standard deviation (based on six measurements) ranged from 0.231 (S21-M) to 0.931 (L36). The difference between the measured average mass and the calculated average mass ranged from 0.05 Da (L33) to 1.00 Da (S21-M) (Supplementary Table 3).

**FIGURE 2:**
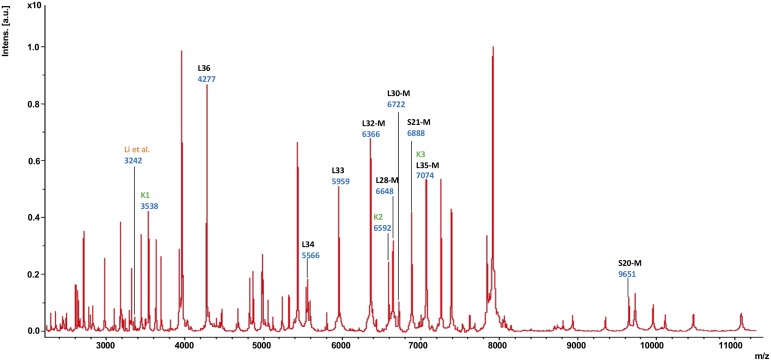
MALDI-TOF mass spectrum of *C. difficile* reference strain 630 (= DSM 27543). In this mass spectrum, all singularly charged biomarkers that were included in the *C. difficile* proteotyping scheme are marked in black; multiply charged ions are not labeled separately. Additionally, the biomarkers used for *C. difficile* subtyping by Li et al. (2018) as well as Kuo et al. (2015) are indicated in orange (“Li”) dark and green (“K1”, “K2”, “K3”), respectively.

Subsequently, the different MS biomarker ions were ascribed to gene products deduced from the genome sequence corresponding to the measured mass taking into account potential post-translational modifications (Figure 2 and Supplementary Table 4). In total, nine singly charged masses of biomarkers were observed in between *m/z* = 4,200 and 9,700 and matched to a specific gene with less than 1.0 Da mass tolerance. The following biomarkers have been identified: RpmJ (L36; 4,277 Da), RpmH (L34; 5,566 Da), RpmG (L33; 5,959 Da), RpmF (L32-M; 6,366 Da), RpmB (L28-M; 6,648 Da), RpmD (L30-M; 6,722 Da), RpsU (S21-M; 6,888 Da), RmpI (L35-M; 7,074 Da), and RpsT (S20; 9,651 Da). As indicated, a post-translational cleavage of the *N*-terminal methionine has been observed in the case of RpmF/L32-M, RpmB/L28-M, RpmD/L30-M, RpsU/S21-M, RmpI/L35-M and RpsT/S20-M (Supplementary Table 4).

### Establishment of an *in silico* Allelic Isoform Database

With the help of 1,312 *C. difficile* sequences deposited in the NCBI database at the time of analysis (June 26th, 2018) we were able to compile a comprehensive list of allelic isoforms for all biomarker ions belonging to the *C. difficile*-specific proteotyping scheme.

Gene sequences deposited for the biomarker isoforms were translated into the respective amino acid sequence and aligned followed by calculation of the protein mass for each individual isoform. The maximum number of biomarker isoforms obtained from the database was 7 for L35-M and S20-M, the minimum number was 3 for L36 and L28-M. Occurrence frequency varied from >99% to a single occurrence of the isoform (Supplementary Table 2). The calculation basis for the frequency of the individual allelic isoforms in Supplementary Table 2 varies from biomarker to biomarker since some of the 1,312 *C. difficile* sequences deposited in the NCBI database were not present as complete closed genomes, and in some cases, the corresponding contigs on which the respective biomarker genes should be located were not available. In case of a single occurrence of an isoform, sequencing errors on the submitter’s side cannot be ruled out. Ignoring all isoforms occurring only 1–3 times in the database, the *C. difficile* proteotyping scheme was mainly based on isoforms of L28-M (two main isoforms), L35-M (four main isoforms), and S20-M (two main isoforms) giving rise to at least 16 proteotyping-derived types. Potentially, there are significantly more proteotyping-derived types to be expected in the population.

### Mass Shifts and Allelic Isoforms in Test Isolate Collection

**FIGURE 3:**
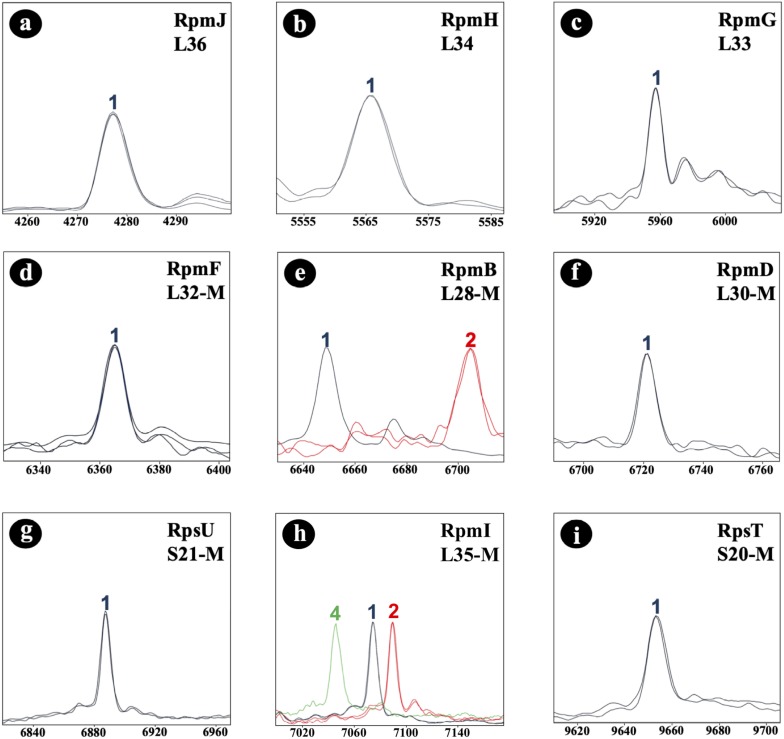
*C. difficile*-specific proteotyping-derived biomarkers **(a–i)**. In order to demonstrate mass differences between allelic isoforms, spectra of representative *C. difficile* isolates of each of the five detected proteotyping-derived types were overlaid. *X*-Axis: mass [Da] charge-1 ratio, scale 200 Da. *Y*-Axis: intensity [10× arbitrary units], spectra were individually adjusted to similar noise in order to improve visualization of peaks with low-intensity. Color codes: the isoform of *C. difficile* reference strain 630 (= DSM 27543) is depicted in blue; red and light green indicate isoforms that differ in their mass from the reference strain 630 (= DSM 27543). Isoforms lacking *N*-terminal methionine are appended with “–M.”

Initially, the *C. difficile* reference strain 630 (= DSM 27543) was analyzed by MALDI-TOF MS. In the study, all mass shift measurements were done with reference to this strain. To identify allelic isoforms the mass shift was compared with the list containing all amino acid sequences. For biomarker RmpI (L35-M) we detected 3 isoforms (7,074.6 Da; 7,090.6 Da; 7,047.5 Da) in the tested isolate cohort, and for biomarker RpmB (L28-M) two isoforms (6,647.8 Da; 6,705.8 Da). RpmJ (L36; 4,277.3 Da), RpmH (L34; 5,565.5 Da), RpmG (L33; 5,959.0 Da), RpmF (L32-M; 6,366.4 Da), RpmD (L30-M; 6,722.9 Da) RpsU (S21-M; 6,889.0 Da), and RpsT (S20-M; 9,651.3 Da) were invariable in the tested isolate cohort (Figure 3), and the biomarker masses corresponded to the respective reference isoforms. Nucleotide and amino acid sequences of the allelic isoforms of biomarkers newly described during the study have been deposited at GenBank. The accession numbers of all biomarkers (nucleotide and amino acid sequences) are listed in Supplementary Table 5.

### Phyloproteomic Analysis

**FIGURE 4:**
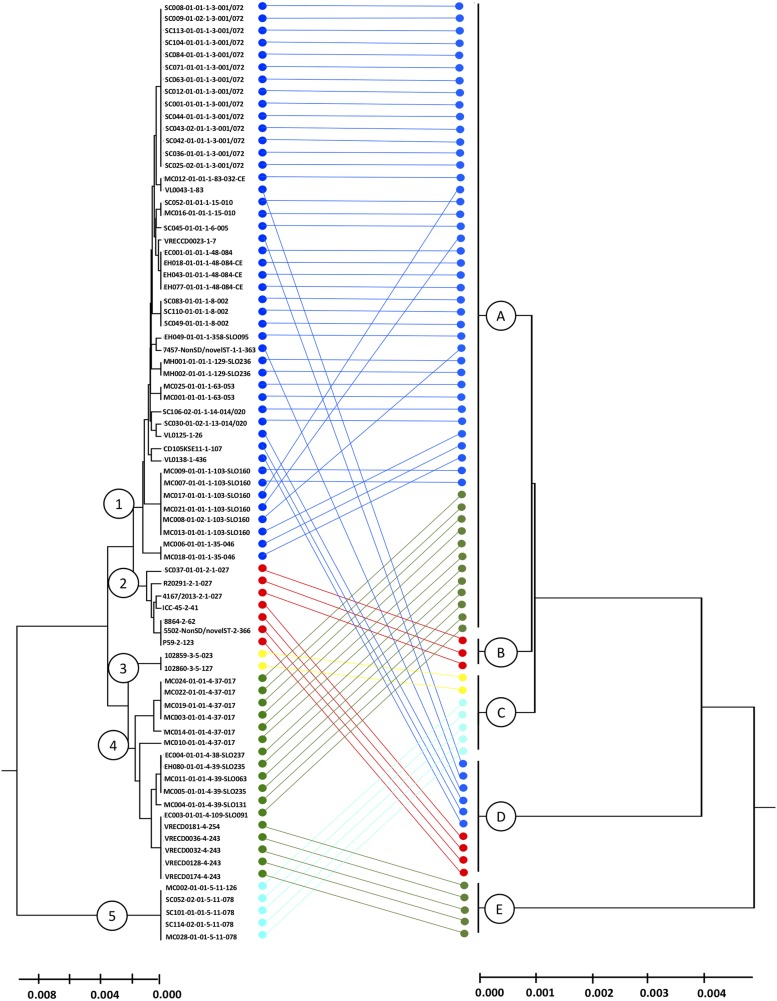
Comparison of MLST- and proteotyping-derived phylogenies. Left tree: MLST-based evolutionary tree using the UPGMA method (maximum composite likelihood method). The isolates of the clades 1-5, indicated by different colors, form coherent clades. Here, the isolates of the clades 1 and 2 as well as the clades 3 and 4 form superclades while the clade 5 remains for itself. Right dendrogram: Proteotyping-derived UPGMA-tree. Here, too, the isolates are arranged in five clades, which, however, do not correspond to the MLST clades. Especially noteworthy is the proteotyping-derived Clade B, which consists of exclusively hypervirulent RT027 isolates, also forming a separate MLST clade (clade 2).

Following the principle of MLST to cluster DNA sequences, the biomarker amino acid sequences of each isolate were concatenated and used to deduce phylogeny by the UPGMA method (conventional clustering algorithm). The combination of amino acid sequences resulted in five different proteotyping-derived types/clades (Figure 4, right dendrogram), here the clades were designated with A–E to prevent confusion with MLST clades, which served as the main comparator (Figure 4, left dendrogram).

The largest proteotyping-derived clade A contained the majority of isolates of MLST clades 1 and 4, while the second largest proteotyping-derived clade D combines isolates of MLST clades 1 and 2.

The smaller proteotyping-derived clades allowed discrimination of more distinctive isolate groups: clade C was formed by all tested *C. difficile* isolates of MLST clade 3 (corresponding to RTs 023 and 127) and MLST clade 5 (corresponding to RTs 078 and 126). Clade E exclusively contained a subgroup of MLST clade 4 isolates, namely RTs 243 and 254. The most interesting finding was that isolates of the highly pathogenic *C. difficile* RT027 formed a unique proteotyping-derived clade (clade B, indicated in red, Figure 3). Since only three isolates belonged to RT027 in the initial test population, which consists of isolates for which a complete genome sequence was available (data not shown), we subsequently (i) checked the genomes from the study of He and coworkers (He et al., 2013) for the presence of alleles encoding for L28-M isoform 2 and L35-M isoform 1 and analyzed (ii) 17 further RT027 isolates as well as (iii) 2 isolates of RT016, 10 isolates of RT176, and 3 isolates of RT153, which are RT027-related ribotypes by MALDI-TOF MS.

The proteotyping-derived type of the RT027 isolates (B) results from the biomarker RpmB/L28-M isoform no. 2 (6,705.8 Da), which corresponds to the amino acid substitution G9D when compared to the *C. difficile* 630 (= DSM 27543) reference isoform, while the biomarker Rpml/L35-M isoform no. 1 (7,074.6 Da) is identical to the *C. difficile* 630 (= DSM 27543) reference isoform.

(i.) In total, we had access to the contigs of 148 genomes of RT027 isolates. Of these 148 datasets, the L28 gene was present in 142 datasets. 100% (142/142) contained the allele encoding the L28-M isoform 2. Of these 142 datasets, 139 contained the gene for L35. 100% (139/139) contained the L35 allele encoding isoform 1. Accordingly, 100% (139/139) of the RT027 genome data sets in which the two biomarker genes were present contained the isoform combination L28-M isoform 2 plus L35-M isoform 1, which had previously been identified as characteristic for RT027.

(ii.) Sanger sequencing of the gene loci of RpmB/L28-M and Rpml/L35-M as well as MALDI-TOF MS confirmed that all 17 additionally tested RT027 isolates also carried the constellation of the RT027 typical isoforms for L28-M and L35-M.

(iii.) MALDI-TOF analysis (that was also reconfirmed by Sanger sequencing), demonstrated that RT153 isolates exhibit the biomarker constellation L28-M isoform 1 and L35-M isoform 1, RT016 isolates exhibit the biomarker constellation L28-M isoform 1 and L35-M isoform 2, and surprisingly RT176 isolates exhibit the biomarker constellation L28-M isoform 2 and L35-M isoform 1. This means that RT016 and RT153 can be distinguished from RT027 by the combination of the two biomarkers L28-M and L35-M, alone. However, the isoform constellation of the biomarkers L28-M and L35-M as well as the remaining seven biomarkers is identical for RT027 and RT176. This means that the ribotypes RT027 and RT176 have the same proteotyping-derived type (B) but can be distinguished as a group from all other tested ribotypes.

## Discussion

In this study, the proteotyping technique previously established for *C. jejuni* (Zautner et al., 2015, 2016) was successfully adapted to *C. difficile.*

The current *C. difficile* proteotyping scheme is based on nine biomarkers, which are exclusively ribosomal proteins. In contrast, the *C. jejuni* proteotyping scheme comprised 19 biomarkers, one being a non-ribosomal protein. The smaller number of detectable biomarkers might be explained by the fact that *C. difficile*, in contrast to *C. jejuni*, is a Gram-positive bacterium and that the Gram-positive cell wall makes it more difficult to release proteins from the cell.

Patterns of post-translational modifications such as the cleavage of *N*-terminal methionine have been shown to be specific for a microbial species (Fagerquist et al., 2006). Six of nine biomarkers in the *C. difficile* mass spectrum showed a cropped methionine, while only six of 19 biomarkers with a cropped methionine were detectable in *C. jejuni* (Zautner et al., 2015). This form of post-translational modification appears thus to be more frequent in the detectable *C. difficile* proteotyping biomarkers than in *C. jejuni*. *N*-terminal methionine is cleaved by the ubiquitous and essential methionine aminopeptidase MAP (Frottin et al., 2006). The *N*-terminal methionine is often removed when the residue at the second position (P1’) in the primary sequence is small and uncharged, i.e., if at position P1’ there is an alanine (A), cysteine (C), glycine (G), proline (P), serine (S), threonine (T), or valine (V). In accordance with this information the biomarkers L32-M (P1’ = A), L28-M (P1’ = A), L30-M (P1’ = A), S21-M (P1’ = S), L35-M (P1’ = P), and S20-M (P1’ = A) are de-methioninated, and the *N*-terminal methionine of L36 (P1’ = K) and L33 (P1’ = R) remains attached. An exception to the aforementioned is L34, which is not de-methioninated although there is a serine at position P1’. It should be noted that the L34 isoforms 3 and 4 have a lysine at position P1’ due to a deletion at position 2.

In our isolate cohort only two biomarkers, L28-M and L35-M, showed mass shifts. The proteotyping-derived phyloproteomic tree (Figure 4) is therefore deduced only from the combination of the two detectable isoforms for L28-M and the three detectable isoforms for L35-M. Of the six (2 × 3) possible combinations of these biomarker isoforms, five combinations or proteotyping-derived types, or clades, were present in the tested isolate cohort. According to our genome analysis, considerably more combinations can be expected in the *C. difficile* population. Especially with the isoforms of the biomarker S20-M seen in the database analysis, 16 or more proteotyping-derived clades can be expected.

Our most relevant finding was the possibility to differentiate a group of isolates formed by the clinically relevant RT027 and the closely related RT176 *C. difficile* isolates from non-RT027/non-RT176 isolates using this proteotyping scheme. While some of our results on the identification of *C. difficile* RT027 strains by MALDI-TOF MS are comparable to those of other studies, most importantly to the one of Reil and coworkers (Reil et al., 2011), also significant differences were detected: We identified a biomarker L28-M isoform lacking *N*-terminal methionine with an average mass of 6,705.8 Da (L28-M isoform no. 2), whereas Reil et al. identified a mass signal at 6,707 Da to be specific for *C. difficile* RT027. The small mass difference (2 Da) of this mass signal in comparison to the one seen in our study may well be attributed due to a difference in calibration. However, Reil and coworkers did not perform further analysis on the gene encoding for the protein indicated by this mass signal, precluding the final confirmation of its identity. Another crucial difference in our study was that we could also demonstrate the corresponding L28-M isoform 1 (6,647.8 Da) to be present in all non-RT027/non-RT176 strains. This finally enabled us to securely differentiate these highly virulent strains from others.

For biomarker L35-M we observed an isoform with an average mass of 7,090.6 Da, likely also shifted by 2 Da in the study by Reil and coworkers (at *m/z* = 7,092 when analyzing RT027 strains). However, they did not consider it to be relevant for differentiation of RT027 from other ribotypes. In our study we found two more L35-M isoforms at 7,074.6 Da and 7,047.5 Da, for which there were no corresponding mass signals in the study of Reil and coworkers. Reil and coworkers also recognized specific markers not only for RT027 but also for the ribotypes RT001 and RT078/126.

Others have also shown the possibility to discriminate between MLST ST37 strains and non-ST37 strains by observing the distribution of two major mass signals at *m/z* = 3,242 and *m/z* = 3,286, respectively (Li et al., 2018). ST37, which mainly corresponds to ribotype 017, has been a dominant ST type in adult *C. difficile* infections in China (Gu et al., 2015; Jin et al., 2016). Indeed, we were able to detect the mass with *m/z* = 3,242 (Figure 2, the mass signal is indicated by “Li”), but unfortunately, we were not able to assign it to a gene, so this mass was excluded from the proteotyping scheme.

However, by means of proteotyping it is not possible to distinguish the higher virulent RT027 isolates from the closely related and also epidemiologically relevant RT176 isolates. But also with other methods it is difficult or even impossible to distinguish these two ribotypes from each other. The PCR-ribotyping profile of RT176 differs from RT027 just by a single band (Krutova et al., 2014) and both Cepheid’s GeneXpert *C. difficile*/Epi and Mobidiag’s Amplidiag^TM^ assays wrongly identify the RT176 as RT027 (Mentula et al., 2015). One possibility to distinguish the two RTs is the detection of a deletion at position 117 in the *tcdC* gene (Krutova et al., 2014). Various isolates of the RT176 could be assigned to the MLST ST1, others to the MLST ST11 (Stabler et al., 2009). While the German reference center for *C. difficile* in Homburg/Saar, Germany, could only detect a total (until May 2019) of 36 isolates of this RT, outbreaks with RT176 have been reported in the Czech Republic and Poland (Nyč et al., 2011). Epidemiological studies in the Czech Republic and Poland have also shown an increased prevalence of RT176, 29% (Krutova et al., 2016) and 14% (Pituch et al., 2015), respectively.

A further study demonstrated that, using a 3-peak pair cluster analysis (*m/z* = K1: 35,38.0/3,545.8; K2: 6,577.9/6,592.8; K3: 7,075.6/7,091.1 Da), it is feasible to detect binary toxin producers of *C. difficile* (Kuo et al., 2015). Mass signals for all three biomarkers were detectable in our recordings (Figure 2). While K2 could not be assigned to any gene and was therefore not included in the proteotyping scheme, K3 corresponded to biomarker L35-M, isoforms no. 1 and 2. K1 likely represented the M + 2H^+^ form (doubly charged biomarker mass) of K3, and was not included. Since the *C. difficile* strain 630 (= DSM 27543) is TcdA^+^, TcdB^+^, CdtA^–^, and CdtB^–^ (Sebaihia et al., 2006; Dannheim et al., 2017), this is in line with the findings of Kuo and his team (Kuo et al., 2015).

The work of Li et al. and Kuo et al. indicates that the potential of proteotyping may probably be even higher, if it were possible to assign further genes to masses of unknown identity.

Cheng and coworkers were able to distinguish *C. difficile* clade 4 isolates from other *C. difficile* isolates based on five different biomarkers (Cheng et al., 2018). A PCA-algorithm was established on the basis of mass spectra of 135 isolates. Subsequently, 25 isolates were used for the validation of the model. The isolates used in the study covered only clades 1,3 and 4 of the eight known *C. difficile* clades.

In comparison to the approach of Cheng and coworkers our proteotyping approach is based on biomarkers of known origin. In our approach the phylogeny is deduced by UPGMA method, not by PCA. Previous studies have shown that PCA results depend on culture conditions as well as time of measurement as it also considers the intensity of peaks. As proteotyping results are not dependent on these factors, they are more reliable (Zautner et al., 2015).

Another relevant study in the context of our study was recently published by Corver and coworkers (Corver et al., 2018). They performed ultrahigh-resolution MALDI-FTICR-MS and identified two mass peaks (*m/z* = 4,927.81 and *m/z* = 5,001.84, a mass change of 74 Da, corresponding to a transition between a single Glycin and Methionine) that allow differentiation of MLST types 1 and 11. The sensitivity and specificity was determined based on the analysis of *C. difficile* sequences in the NCBI database. Both mass peaks could be assigned to two different isoforms of an uncharacterized protein. According to a BLAST-search the peptide could be a fragment of the protein CDIF630_01208. Thus, this protein is a potential candidate for the extension of the proteotyping scheme. However, the detection of this biomarker requires the ultrahigh-resolution MALDI-FTICR-MS, a technique not available in diagnostic-microbiological routine. Thus, this biomarker is currently not ready for integration in our proteotyping scheme. Additionally, an enrichment procedure has been developed that has been demonstrated to enrich the peptide MLST-1 markers sufficiently, which would facilitate processing in a normal MALDI-TOF workflow. However, this enrichment procedure yielded inconsistent results for MLST-11 and MLST-15 isolates.

Lastly, a method designated high molecular weight (HMW) typing has been shown to allow *C. difficile* typing. In this method, a protein profile in the range between 30 and 50 kDa is analyzed. Although the method was less discriminatory than PCR-ribotyping, results have been obtained fast, simple and cost-effective (Rizzardi and Åkerlund, 2015; Ortega et al., 2018). For this method, too, it must be acknowledged that special mass spectrometric equipment must be available which goes beyond the current standards of routine diagnostics.

In comparison to sequence-based methods such as MLST or MLVA (Multiple loci variable-number tandem repeat analysis), the discriminatory depth of proteotyping is limited. Compared to whole genome sequence based methods, it is not feasible to show the clonality of isolates by proteotyping. Therefore, the practicability of proteotyping depends on the specific epidemiological question. To improve discriminatory capacity of our method, future studies should focus on the identification of additional biomarkers and the assignment to the respective gene loci. Furthermore, it should be aimed to extend the recordable mass spectrum. The development of a user-friendly bioinformatic solution and implementation into the standard software of the manufacturer could further facilitate the application of the technique in daily routine diagnostics.

## Conclusion

The crucial difference between proteotyping and other MALDI-TOF MS-based techniques is that in case of proteotyping the protein isoform behind the peaks is known. Where other methods consider presence or absence of single masses as well as the peak intensity, in proteotyping differentiation is achieved on the basis of an exclusive combination of known, and ideally genetically verified, biomarker masses. This is achieved by genomic analysis of genes encoding ribosomal proteins of a species. Mutations resulting in changes of the amino acid sequences result in peak shifts in the MALDI-TOF spectra.

Our study shows that our formal *C. difficile*-specific 9 biomarker proteotyping scheme is discriminatory to differentiate between strains of RT027/176 and non-RT027/176 strains. While both methods are not congruent, the discriminatory depth of *C. difficile* proteotyping corresponds at least to the MLST clade classification, but is potentially also higher. More genome sequences resulting in more isoforms and therewith more proteotyping-derived types would improve the discriminatory depth of the method significantly. In addition to the number of isoforms, more precise mass spectrometric methods can also be used to increase the number of biomarkers and thus the discriminatory depth.

Since immediate responses are highly important in case of disease outbreaks (mainly corresponding to RT027 and RT176), our method offers a fast, accurate and inexpensive initial diagnostic tool that can provide indications of RT027/176 outbreaks.

In addition, it should be noted that RT027 and RT176 are only two of the different RTs associated with more severe disease and increased mortality. In consequence RT is not an unequivocal predictor of a severe CDI (Walk et al., 2012; Aitken et al., 2015). In this context, the timely determination of the RT as an infection control tool has yet to prove its effectiveness (Scardina et al., 2015; Aktories et al., 2017).

## Ethics Statement

Ethical clearance for the analysis was obtained from the Ethics Committee of the University Medical Center Göttingen, Germany. No humans, animals, or personalized data were used for this study.

## Author Contributions

ME performed the data analysis, PCR and Sanger sequencing, and wrote the manuscript. FJ created the isoform database. TR and JO performed the sequencing. MR performed the ribotyping. FL the performed bacterial culture and recorded the mass spectra. OZ, PC, FB, and RK isolated, collected, and identified all *C. difficile* isolates. WB and AZ performed the WGS sequence analysis. UG, OB, and AZ conceived and designed the experiments, performed the data analysis, and wrote the manuscript including figures. AZ performed the MLST analysis and calculated the taxonomic dendrograms. All authors have proofread the manuscript and agreed on publication.

## Conflict of Interest Statement

The authors declare that the research was conducted in the absence of any commercial or financial relationships that could be construed as a potential conflict of interest.

## Footnotes

1 http://web.expasy.org/compute_pi/
