# Supplementary material for: Proteotyping of Clostridioides difficile as Alternate Typing Method to Ribotyping Is Able to Distinguish the Ribotypes RT027 and RT176 From Other Ribotypes
Source: Front Microbiol. 2019 Sep 10;10:2087. doi: 10.3389/fmicb.2019.02087 (PMC6747054; doi:10.3389/fmicb.2019.02087)
Supplement: Supplementary file 1 [file Table_1.docx]

**Supplementary Table 1: List of *Clostridioides difficile* Isolates**

| **Isolate** | **Clade** | **MLST-ST** | **PCR-Ribotype** | **Origin** |
| --- | --- | --- | --- | --- |
| EC001-01-01 (DSM 28666) | 1 | 48 | 084 (CE) | Ghana |
| EC003-01-01 (DSM 28669) | 4 | 109 | SLO091 | Ghana |
| EH018-01-01 | 1 | 48 | 084 (CE)  084 (CE) | Ghana |
| EH043-01-01 | 1 | 48 | 084 (CE) | Ghana |
| EH077-01-01 | 1 | 48 | 084 (CE) | Ghana |
| EH080-01-01 | 4 | 39 | SLO235 | Ghana |
| MC006-01-01 | 1 | 35 | 046 | Indonesia |
| MC009-01-01 | 1 | 103 | SLO160 | Indonesia |
| MC010-01-01 | 4 | 37 | 017 | Indonesia |
| MC011-01-01 | 4 | 39 | SLO063 | Indonesia |
| MC012-01-01 | 1 | 83 | 032 (CE) | Indonesia |
| MC014-01-01 | 4 | 37 | 017 | Indonesia |
| SC025-02-01 | 1 | 3 | 001/072 | Germany |
| SC036-01-01 | 1 | 3 | 001/072 | Germany |
| SC042-01-01 | 1 | 3 | 001/072 | Germany |
| SC043-02-01 | 1 | 3 | 001/072 | Germany |
| SC044-01-01 | 1 | 3 | 001/072 | Germany |
| EC004-01-01 (DSM 28670) | 4 | 38 | SLO237 | Ghana |
| EH049-01-01 | 1 | 358 | SLO095 | Ghana |
| MC004-01-01 | 4 | 39 | SLO131 | Indonesia |
| MC007-01-01 | 1 | 103 | SLO160 | Indonesia |
| MC017-01-01 | 1 | 103 | SLO160 | Indonesia |
| MC018-01-01 | 1 | 35 | 046 | Indonesia |
| MC021-01-01 | 1 | 103 | SLO160 | Indonesia |
| MC025-01-01 | 1 | 63 | 053 | Indonesia |
| MH001-01-01 | 1 | 129 | SLO236 | Indonesia |
| MH002-01-01 | 1 | 129 | SLO236 | Indonesia |
| SC001-01-01 | 1 | 3 (2) | 001/072 | Germany |
| SC012-03-01 | 1 | 3 | 001/072 | Germany |
| SC063-01-01 | 1 | 3 | 001/072 | Germany |
| SC083-01-01 | 1 | 8 | 002 | Germany |
| MC001-01-01 | 1 | 63 | 053 | Indonesia |
| MC002-01-01 | 5 | 11 | 126 | Indonesia |
| SC052-02-01 (DSM 29747) | 5 | 11 | 078 | Germany |
| SC052-01-01 (DSM 29688) | 1 | 15 | 010 | Germany |
| SC045-01-01 | 1 | 6 | 005 | Germany |
| SC071-01-01 | 1 | 3 | 001/072 | Germany |
| SC084-01-01 | 1 | 3 | 001/072 | Germany |
| SC101-01-01 | 5 | 11 | 078 | Germany |
| SC104-01-01 | 1 | 3 | 001/072 | Germany |
| SC106-02-01 | 1 | 14 | 014/020 | Germany |
| SC110-01-01 | 1 | 8 | 002 | Germany |
| SC113-01-01 | 1 | 3 | 001/072 | Germany |
| SC114-02-01 | 5 | 11 | 078 | Germany |
| MC016-01-01 | 1 | 15 | 010 | Indonesia |
| DSM 102859 | 3 | 5 | 023 | Germany |
| DSM 102860 | 3 | 5 | 127 | Germany |
| MC003-01-01 (DSM 29627) | 4 | 37 | 017 | Indonesia |
| MC005-01-01 (DSM 29629) | 4 | 39 | SLO235 | Indonesia |
| MC008-01-02 | 1 | 103 | SLO160 | Indonesia |
| MC013-01-01 | 1 | 103 | SLO160 | Indonesia |
| MC019-01-01 | 4 | 37 | 017 | Indonesia |
| 4167/2013 1 (DSM 27638) | 2 | 1 | 027 | Germany |
| MC022-01-01 | 4 | 37 | 017 | Indonesia |
| MC024-01-01 | 4 | 37 | 017 | Indonesia |
| MC028-01-01 | 5 | 11 | 078 | Indonesia |
| R20291 (DSM 27147) | 2 | 1 | 027 | Great Britain |
| SC008-01-01 | 1 | 3 | 001/072 | Germany |
| SC009-01-02 | 1 | 3 | 001/072 | Germany |
| SC030-01-02 | 1 | 13 | 014/020 | Germany |
| SC037-01-01 (DSM 28196) | 2 | 1 | 027 | Germany |
| SC049-01-01 | 1 | 8 | 002 | Germany |
| VL0125 | 1 | 26 | 140 | Germany |
| 8864 | 2 | 62 | non-specific | Germany |
| VRECD0023 | 1 | 7 | 026 | Germany |
| CD105KSE11 | 1 | 107 | non-specific | Germany |
| VL0043 | 1 | 83 | non-specific | Germany |
| VL0138 | 1 | 436 | non-specific | Germany |
| ICC-45 | 2 | 41 | 106/194/321 | Germany |
| 7457-NonSD/novelST | 1 | 363 | non-specific | Germany |
| 5502-NonSD/novelST | 2 | 366 | non-specific | Germany |
| P59 | 2 | 123 | non-specific | Germany |
| VRECD0036 | 4 | 243 | non-specific | Germany |
| VRECD0032 | 4 | 243 | non-specific | Germany |
| VRECD0181 | 4 | 254 | non-specific | Germany |
| VRECD0174 | 4 | 243 | non-specific | Germany |
| VRECD0128 | 4 | 243 | non-specific | Germany |
| 5285-2016 rau | 2 | 1 | 027 | Germany |
| 5285-2016 glatt | 2 | 1 | 027 | Germany |
| 3067-2018 | 2 | 1 | 027 | Germany |
| 2912-2018 | 2 | 1 | 027 | Germany |
| 3496-2018 | 2 | 1 | 027 | Germany |
| HK2-2016 | 2 | 1 | 027 | Germany |
| HK078 | 2 | 1 | 027 | Germany |
| OZ-2016 | 2 | 1 | 027 | Germany |
| 2829-2018 | 2 | 1 | 027 | Germany |
| 2990-2016 | 2 | 1 | 027 | Germany |
| 6960-2015 | 2 | 1 | 027 | Germany |
| 1141-2014a | 2 | 1 | 027 | Germany |
| 1141-2014b | 2 | 1 | 027 | Germany |
| 3461-2018 | 2 | 1 | 027 | Germany |
| 8574-2016 (1) | 2 | 1 | 027 | Germany |
| 8574-2016 (2) | 2 | 1 | 027 | Germany |
| 2667-2018 | 2 | 1 | 027 | Germany |
| 1103365 | - | - | 176 | Germany |
| 1104006 | - | - | 176 | Germany |
| 1105000 | - | - | 176 | Germany |
| 1105010 | - | - | 176 | Germany |
| 1105017 | - | - | 176 | Germany |
| 1730076 | - | - | 176 | Germany |
| 1732914 | - | - | 176 | Germany |
| 1739826 | - | - | 176 | Germany |
| 1740542 | - | - | 176 | Germany |
| 1734994 | - | - | 176 | Germany |
| 1734935 | - | - | 153 | Germany |
| 1737590 | - | - | 153 | Germany |
| 1743607 | - | - | 153 | Germany |
| 07-00059 | - | - | 016 | Germany |
| 08-00313 | - | - | 016 | Germany |
