# Supplementary material for: Proteotyping of Clostridioides difficile as Alternate Typing Method to Ribotyping Is Able to Distinguish the Ribotypes RT027 and RT176 From Other Ribotypes
Source: Front Microbiol. 2019 Sep 10;10:2087. doi: 10.3389/fmicb.2019.02087 (PMC6747054; doi:10.3389/fmicb.2019.02087)
Supplement: Supplementary file 2 [file Table_2.docx]

**Supplementary Table 2. Overview of all isoforms included in the *C. difficile* proteotyping scheme**

**Link to *C. difficile* strain 630 genome:** **https://www.ncbi.nlm.nih.gov/nuccore/CP010905.2**

| **Locus** | **Full name / product (ORF Locus tag in *C. difficile* 630)** | **calc. Mass [Da]** | | **Frequency in database** |
| --- | --- | --- | --- | --- |
| **RpmJ L36 CDIF630****_00164 / AJP09789.1** | | | | |
| sequence | MKVRPSVKPICEKCKVIKRKGKVMVICENPKHKQKQG* (37 aa) | | | |
| 1 * | *C. difficile* 630 reference isoform | 4277.34 Da | ±0.00 Da | 99.610 % (1278/1283) |
| 2 | MKVRPSVKPMCEKCKIIKRKGKVMVICENPKHKQKQG (I10M+ V16I) | 4309.40 Da | +32.06 Da | 0.233 % (3/1283) |
| 3 | MKVRPSVKPICDKCRIIKRKGRVMVICENPKHKQRQG (E12D+K15R+ V16I+ K22R+K35R) | 4361.38 Da | +84.04 Da | 0.077 % (1/1283) |

| **RpmH L34 CDIF630_04011 / AJP13465.1** | | | | |
| --- | --- | --- | --- | --- |
| sequence | MSKRTYQPKKRQRSKEHGFRKRMKTSNGRNVLKRRRAKGRNRLTH (45 aa) | | | |
| 1 * | *C. difficile* 630 reference isoform | 5565.53 Da | ±0.00 Da | 99.694 % (1305/1309) |
| 2 | MSKRTYQPKKRQRSKEHGFRKRMKTSNGRNALKRRRAKGRNRLTH (V31A) | 5537.48 Da | -28.05 Da | 0.076 % (1/1309) |
| 3 | M-KRTYQPKKRQRKKEHGFRKRMKTSNGRNVLKRRRAKGRNRLTH (S2Del+R14K) | 5519.55 Da | -45.98 Da | 0.076 % (1/1309) |
| 4 | M-KRTYQPKKRQRKKEHGFRKRMKTSNGRNVLKRRRSKGRNRLTH (S2Del+ R14K+ A37S) | 5535.55 Da | -29.98 Da | 0.153 % (2/1309) |

| **RpmG L33 CDIF630_00123 / AJP09749.1** | | | | |
| --- | --- | --- | --- | --- |
| sequence | MRVKVTLACTECKQRNYNTTKNKKNNPDRIELQKYCRFCKKHTTHKETK (49 aa) | | | |
| 1 * | *C. difficile* 630 reference isoform | 5958.95 Da | ±0.00 Da | 99.533 % (1279/1285) |
| 2 | MRVKVTLACTECKQRNYNTTKNKKNNPDRIELQKYCRFCKKHATHKETK (T43A) | 5928.93 Da | -30.02 Da | 0.156 % (2/1285) |
| 3 | MRVKVTLACKECKQRNYNTTKNKKNNPDRIELQKYCRFCKKHTTHKETK (T10K) | 5986.02 Da | +27.07 Da | 0.077 % (1/1285) |
| 4 | MRVKVTLACSECKQRNYNTTKNKKNNPDRIELNKYCKFCKKHTTHKETK (T10S+ Q33N+ R37K) | 5902.89 Da | -56.06 Da | 0.233 % (3/1285) |

| **RpmF L32-M CDIF630_01325 / AJP10868.1** | | | | |
| --- | --- | --- | --- | --- |
| sequence | (M)AVPKRKTSKSNTKMRRAANSKMEATGFVSCPQCHEPKLPHRVCPDCGYYKGKEVVSK (58 aa) | | | |
| 1 * | *C. difficile* 630 reference isoform | 6366.44 Da | ±0.00 Da | 99.618 % (1303/1308) |
| 2 | (M)AVPKRKTSKSNTKMRRGANSKMEATGFVSCPQCHEPKLPHRVCPDCGYYKGKEVVSK (A18G) | 6352.41 Da | -14.03 Da | 0.076 % (1/1308) |
| 3 | (M)AVPKRKTSKSNTKMRRAAKSKMEATGFVSCPQCHEPKLPHRVCPDCGYYKGKEVVSK (N20K) | 6380.51 Da | +14.07 Da | 0.076 % (1/1308) |
| 4 | (M)AVPKRKTAKSKTKMRRAANSKMTATGFVECPQCHEPKLPHRVCPDCGHYKGKEIVSE (S9A+N12K+E24T+S30E+Y49H+V55I+K58E) | 6367.47 Da | +1.03 Da | 0.229 % (3/1308) |

| **RpmB L28-M CDIF630_02814 / AJP12309.1** | | | | |
| --- | --- | --- | --- | --- |
| sequence | (M)AKVCSVCGKGKVSGNQVSHSNKHNKRTWSANLRSVRAIIDGAPKRVKVCTRCLRSGKIERA (62 aa) | | | |
| 1 * | *C. difficile* 630 reference isoform | 6647.77 Da | ±0.00 Da | 84.037 % (1095/1303) |
| 2 * | (M)AKVCSVCDKGKVSGNQVSHSNKHNKRTWSANLRSVRAIIDGAPKRVKVCTRCLRSGKIERA (G9D) | 6705.81 Da | +58.04 Da | 15.886 % (207/1303) |
| 3 | (M)AKVCSVCGKGKVSGNQVSHSNKHNKRTWSANLRSVRAIIYGAPKRVKVCTRCLRSGKIERA (D41Y) | 6695.86 Da | +48.09 Da | 0.076 % (1/1303) |

| **RpmD L30-M CDIF630_00157 / AJP09782.1** | | | | |
| --- | --- | --- | --- | --- |
| sequence | (M)AKLQIKLVRSVIGTTPNQKKNVEALGLRKREQVVVKEDNAQTRGMINKVSHLLEVTEIAE (61 aa) | | | |
| 1 * | *C. difficile* 630 reference isoform | 6722.86 Da | ±0.00 Da | 99.376 % (1274/1282) |
| 2 | (M)AKLQIKLVRSIIGTTPNQKKNVEALGLRKREQVVVKEDNAQTRGMINKVSHLLEVTEIAE (V12I) | 6736.88 Da | +14.02 Da | 0.156 % (2/1282) |
| 3 | (M)AKLQIKLVRSVIGTTPNQKKNVEALGLRKREQVVVKEDNAQTRGMIDKVSHLLEVTEIAE (N48D) | 6723.84 Da | +0.98 Da | 0.078 % (1/1282) |
| 4 | (M)AKLQIKLVRSVIGTTPNQKKNVEALGLRKREQVVIKEDNAQTRGMISKVSHLLEVTEIAE (V36I+N48S) | 6709.86 Da | -13.00 Da | 0.156 % (2/1282) |
| 5 | (M)AKLQIKLVRSTIGTTPNQRKNVEALGLTKREQVVVKEDNAQMRGMISKVSHLVEVTEITE (V12T+K20R+R29T+T43M+N48S+L54V+A60T) | 6716.82 Da | -6.04 Da | 0.078 % (1/1282) |
| 6 | (M)AKLQIKLVRSTIGTTPNQRKNVEALGLTKREQVVVKEDNAQMRGMIAKVSHLVEVTEITE  (V12T+K20R+R29T+T43M+N48A+L54V+A60T) | 6700.82 Da | -22.04 Da | 0.156 % (2/1282) |

| **RpsU S21-M CDIF630_02691 / AJP12193.1** | | | | |
| --- | --- | --- | --- | --- |
| sequence | (M)SEVRVRENETLDSALRRFKRQCAMSGIMSEVRKREHYDKPSVKRKKKAEAARRKNAKK (59 aa) | | | |
| 1 * | *C. difficile* 630 reference isoform | 6889.00 Da | ±0.00 Da | 99.695 % (1308/1312) |
| 2 | (M)SEVRVRENETLDSALRRFKRQCAMSGIMSEVRKREHYDKPSVKRKKKAEAARRKNAKR (K59R) | 6917.02 Da | +28.02 Da | 0.152 % (2/1312) |
| 3 | (M)SEVRVRENESLDSALRRFKRQCAMSGIMSEVRKREHYDKPSVKRKKKAEAARRKNAKR (T11S+K59R) | 6902.99 Da | +13.99 Da | 0.076 % (1/1312) |
| 4 | (M)SEVRVRENETLDSALRRFKRQCAMSGIMSEVRKREHYDKSSVKRKKKAEAARRKNAKK (P41S) | 6878.97 Da | -10.03 Da | 0.076 % (1/1312) |

| **Rpml L35-M CDIF630_00799 / AJP10362.1** | | | | |
| --- | --- | --- | --- | --- |
| sequence | (M)PKMKTHRGAAKRLKKTGTGKLKRAKAFKKHILTKKSAKTKMNLRKSTLVSDGDAKRIAQLLPY (64 aa) | | | |
| 1 * | *C. difficile* 630 reference isoform | 7074.60 Da | ±0.00 Da | 89.397 % (1172/1311) |
| 2 * | (M)PKMKTHRGAAKRLKKTGTGKLKRAKAYKKHILTKKSAKTKMNLRKSTLVSDGDAKRIAQLLPY (F28Y) | 7090.59 Da | +15.99 Da | 5.645 % (74/1311) |
| 3 | (M)PKMKTHRGAAKRLKKTGTGKLKRAKAFKRHKLTKKSAKNKMNLRKSTLVSDGDAKRIAQLLPY (K30R+I32K+T40N) | 7130.62 Da | +56.02 Da | 0.076 % (1/1311) |
| 4 * | (M)PKMKTHRGAAKRLKKTGTGKLKRAKAFKRHKLTGKSAKTKMDLRKSTLVSDGDAKRIAQLLPY (K30R+I32K+T35G+N43D) | 7047.49 Da | -27.11 Da | 3.967 % (52/1311) |
| 5 | (M)PKMKSHRGAAKRLKKTGTGKLKRPKAFKRHKLTKKSAKNKMNLRKSTLVSDGDAKRIAQLLPY (A25P+I32K+T40N) | 7142.63 Da | +68.03 Da | 0.686 % (9/1311) |
| 6 | (M)PKMKTHRGAAKRFKKTGSGKLKRAKAFKSHILTKKSAKTKMNLRQSAIVTKGDAERIAQLLPY (L14F+T19S+K30S+K46Q+T48A+L49I+S51T+D52K+K56E) | 7051.47 Da | -23.13 Da | 0.076 % (1/1311) |
| 7 | (M)PKMKTHRGAAKRFKKTGSGKLKRAKAFKSHILTKKSPKTKMNLRQSAIVSKGDAERIAQLLPY (L14F+T19S+K30S+A38P+K46Q+T48A+L49I+D52K+K56E) | 7063.48 Da | -11.12 Da | 0.153 % (2/1311) |

| **RpsT S 20-M CDIF630_02719 / AJP12221.1** | | | | |
| --- | --- | --- | --- | --- |
| sequence | (M)ANIKSAKKRISVIEKKTALNRVRKSQIKTAIRRFEDAVAAGNREDAVAKFQYAQKRIYQVASKGTIHKNAAARKVAKLAQKLNGMNA (88 aa) | | | |
| 1 * | *C. difficile* 630 reference isoform | 9651.30 Da | ±0.00 Da | 98.926 % (1290/1304) |
| 2 | (M)ANIKSAKKRISVIEKKTALNRVRKSQIKTAIRRFEDAIAAGNREDAVAKFQYAQKRIYQVASKGTIHKNAAARKVAKLAQKLNGMNA (V39I) | 9665.33 Da | +14.03 Da | 0.077 % (1/1304) |
| 3 | (M)ANIKSAKKKISVIEKKTALNRVRKSQIKTAIRRFEDAVAAGNREDAVAKFQYAQKRIYQVASKGTIHKNAAARKVAKLAQKLNGMNA (R10K) | 9623.29 Da | -28.01 Da | 0.077 % (1/1304) |
| 4 | (M)ANIKSAKKRISVIEKKTALNRVRKSQIKTAIRRFEDAVAAGNREDAVAKFQYAQKRIYQVASKGTIHKNAEARKVAKLAQKLNGMNA (A72E) | 9709.34 Da | +58.04 Da | 0.077 % (1/1304) |
| 5 | (M)ANIKSAKKRIGVIEKKTALNRVRKSQIKTAIRRFEDAVAAGNREDAVAKFQYAQKRIYQVASKGTIHKNAAARKVAKLAQKLNGMNA (S12G) | 9621.28 Da | -30.02 Da | 0.460 % (6/1304) |
| 6 | (M)ANIKSAKKRIGVIEKKTALNKVRKSQIKTAIRRFEDAVAAGNREDAVAKFQYAQKRIYQVASKGTIHKNAAARKVAKLAQKLNGMNA (S12G+R22K) | 9593.26 Da | +58.04 Da | 0.077 % (1/1304) |
| 7 | (M)ANIKSAKKRIKVIDKKTALNKARKSQLKTAIRRFDEAVTAGNVEEATARFQYAQKRIYQVASKGTIHKNAAARKVAKLAQKLNAMNA (S12K+E15D+V23A+I28L+E36D+D37E+A40T+R44V+D46E+V48T+K50R+G85A) | 9653.32 Da | +2.02 Da | 0.230% (3/1304) |
