# Supplementary material for: Proteotyping of Clostridioides difficile as Alternate Typing Method to Ribotyping Is Able to Distinguish the Ribotypes RT027 and RT176 From Other Ribotypes
Source: Front Microbiol. 2019 Sep 10;10:2087. doi: 10.3389/fmicb.2019.02087 (PMC6747054; doi:10.3389/fmicb.2019.02087)
Supplement: Supplementary file 3 [file Table_3.DOCX]

**Supplementary Table 3. Comparative presentation of actual measured mass and calculated monoisotopic and average masses**

| **Biomarker** | **Isoform** | **Measured Mass**  **[Da]** | **Standard deviation** | ∆ **Measured mass/**  **Average mass** | **Monoisotopic mass**  **[Da] calc.** | **Average Mass [Da] calc.** |
| --- | --- | --- | --- | --- | --- | --- |
| L36 | Isoform 1 | 4277 | 0.931 | 0.34 | 4274.40 | 4277.34 |
| L34 | Isoform 1 | 5566 | 0.622 | 0.47 | 5562.13 | 5565.53 |
| L33 | Isoform 1 | 5959 | 0.638 | 0.05 | 5955.05 | 5958.95 |
| L32-M | Isoform 1 | 6366 | 0.468 | 0.44 | 6362.21 | 6366.44 |
| L28-M | Isoform 1 | 6648 | 0.728 | 0.23 | 6643.58 | 6647.77 |
| L28-M | Isoform 2 | 6705 | 0.445 | 0.81 | 6701.58 | 6705.81 |
| L30-M | Isoform 1 | 6722 | 0.512 | 0.86 | 6718.78 | 6722.86 |
| S21-M | Isoform 1 | 6888 | 0.231 | 1.00 | 6884.74 | 6889.00 |
| L35-M | Isoform 1 | 7074 | 0.409 | 0.60 | 7070.18 | 7074.60 |
| L35-M | Isoform 2 | 7091 | 0.721 | 0.41 | 7086.17 | 7090.59 |
| L35-M | Isoform 4 | 7047 | 0.793 | 0.49 | 7043.11 | 7047.49 |
| S20-M | Isoform 1 | 9651 | 0.676 | 0.30 | 9645.46 | 9651.30 |
