# Supplementary material for: Proteotyping of Clostridioides difficile as Alternate Typing Method to Ribotyping Is Able to Distinguish the Ribotypes RT027 and RT176 From Other Ribotypes
Source: Front Microbiol. 2019 Sep 10;10:2087. doi: 10.3389/fmicb.2019.02087 (PMC6747054; doi:10.3389/fmicb.2019.02087)
Supplement: Supplementary file 5 [file Table_5.DOCX]

**Supplementary Table 5. GenBank Accession Numbers of C. difficile-specific proteotyping biomarker isoforms.**

| **Biomarker** | **Isoform** | **Accession No.** | **Locus Tag** | **Protein ID** |
| --- | --- | --- | --- | --- |
| L36 | 1 | CP012309.1 | CDIF28668_00164 | AXU70178.1 |
| L34 | 1 | CP012309.1 | CDIF28668_03894 | AXU73745.1 |
| L33 | 1 | CP012309.1 | CDIF28668_00123 | AXU70138.1 |
| L32-M | 2 | CP012309.1 | CDIF28668_01177 | AXU71120.1 |
| L28-M | 1 | CP012309.1 | CDIF28668_02739 | AXU72630.1 |
| L28-M | 2 | CP012320.1 | CDIF28196_02660 | AXU57997.1 |
| L30-M | 1 | CP012309.1 | CDIF28668_00157 | AXU70171.1 |
| S21-M | 1 | CP012309.1 | CDIF28668_02617 | AXU72515.1 |
| L35-M | 1 | CP012309.1 | CDIF28668_00758 | AXU70712.1 |
| L35-M | 2 | CP020378.1 | CDIF102859_00833 | AXU26679.1 |
| L35-M | 4 | MK422610 |  |  |
| S20-M | 2 | CP012309.1 | CDIF28668_02644 | AXU72542.1 |
